# Comparative analysis of complete nucleotide sequence of porcine reproductive and respiratory syndrome virus (PRRSV) isolates in Thailand (US and EU genotypes)

**DOI:** 10.1186/1743-422X-6-143

**Published:** 2009-09-16

**Authors:** Alongkorn Amonsin, Roongtham Kedkovid, Suphasawatt Puranaveja, Piya Wongyanin, Sanipa Suradhat, Roongroje Thanawongnuwech

**Affiliations:** 1 Faculty of Veterinary Science, Chulalongkorn University, Henri-Dunant Road, Patumwan, Bangkok 10330, Thailand

## Abstract

**Background:**

Porcine reproductive and respiratory syndrome virus (PRRSV) is a causative agent of Porcine Reproductive and Respiratory Syndrome (PRRS). In this study, the complete nucleotide sequences of the selected two Thai PRRSV isolates, EU (01CB1) and US (01NP1) genotypes were determined since both isolates are the Thai prototypes.

**Results:**

01CB1 and 01NP1 contain 14,943 and 15,412 nucleotides, respectively. The viruses compose 2 untranslated regions (5' UTR and 3' UTR) and 8 open reading frames (ORFs) designated as ORF1a, ORF1b and ORF2-7. Phylogenetic analysis of full length of the viruses also showed that the 01CB1 and 01NP1 were grouped into the EU and US genotype, respectively. In order to determine the genetic variation and genetic relatedness among PRRSV isolates, the complete nucleotide sequences of PRRSV isolated in Thailand, 01CB1 and 01NP1 were compared with those of 2 EU strains (Lelystad, and EuroPRRSV), 6 US strains (MLV, VR2332, PA8, 16244B, SP and HUN4). Our results showed that the 01CB1 genome shares approximately 99.2% (Lelystad) and 95.2% (EuroPRRSV) nucleotide identity with EU field strains. While, the 01NP1 genome has 99.9% nucleotide identity with a live vaccine strain (MLV) and 99.5% and 98.5% nucleotide identity with 2 other US isolates, VR2332 and 16244B, respectively. In addition, ORF5 nucleotide sequences of 9 PRRS viruses recovered in Thailand during 2002-2008 were also included in this study. Phylogenetic analysis of ORF5 showed high similarity among EU and US genotypes of the recent Thai PRRS viruses (2007-2008 viruses) with 01CB1 and 01NP1.

**Conclusion:**

Overall, the results suggested that the Thai EU isolate (01CB1) may evolve from the EU prototype, Lelystad virus, whereas the Thai US isolate (01NP1) may originate and evolve from the vaccine virus or its derivatives. Interestingly, the US-MLV vaccine was not available in the Thai market in 2001. The Vaccine-like virus might have persisted in the imported pigs or semen and later spread in the Thai swine industry. This report is the first report of complete nucleotide sequences of the Thai PRRS viruses both EU and US genotypes.

## Background

Porcine reproductive and respiratory syndrome virus (PRRSV) belonging to the genus *Arterivirus *in the family *Arteriviridae *in the order *Nidovirales *is a major swine virus causing economic losses in the swine industry worldwide including Thailand. Porcine reproductive and respiratory syndrome (PRRS) was first evident in the North American countries in 1987 and later in the European countries in 1990 [1]. In Thailand, PRRSV was first isolated in 1996 [2] but was serologically evident since 1989. Our previous report demonstrated that in Thailand both US and EU genotypes exist and sequential analysis of ORF5 gene confirmed genetic variation of Thai PRRS viruses [3].

Full-length genome sequences of PPRSV are essential and have been used for gene functional study, pathogenesis study, and evolutionary study as well as vaccine development of the virus. Full-length sequences of several PRRSV both US and EU genotypes are available in the public database. For example, the US prototype (VR2332) [4] and European prototype (LV) [5] are well characterized. The US genotype strains including the US field strains (16244B) [6], the US-MLV vaccine (MLV) [7], Canadian field strain (PA8) [8], Asian vaccine strain (SP) [9] and Asian field strain (BJ-4 and HUN4) [10] are available in the database. In addition, full-length sequences of EU genotype strain (EuroPRRSV) [11] was also identified. Phylogenetic analysis and full-length sequence comparison of the prototype viruses revealed that US and EU strains share approximately 63% nucleotide homology [6]. It has been known that ORF1a is relatively high variable, while ORF1b is more conserved among US and EU genotypes. Recent example is that the variation of ORF1a (multiple deletions in Nsp2 region) related to atypical virulence of PRRS in China [10,12]. The structural protein encoding genes (ORFs2-7) are 20% (3 kb) in length of the genome. Out of 6 structural genes, ORF5 and ORF7 have been widely characterized and used to study the genetic diversity of the viruses in several reports [13-17]

In this present study, we described the genetic comparison of full-length sequences of two Thai PRRSV prototypes of both EU (01CB1) and US (01N1) genotypes. ORF5 nucleotide sequences of 9 PRRS viruses recovered during 2002-2008 were also included in the analysis. Overall, 01CB1 closely related to the Lelystad virus (99.2%) and EuroPRRSV (95.2%). On the other hand, the 01NP1 genome was similar to the US-MLV, VR2332 and 16244B at 99.9%, 99.5% and 98.5% identity, respectively. The availability of complete genome sequences of Thai PRRSV is essential and useful for the evolution study of PRRSV as well as the development of infectious clones or vaccines in the future.

## Results

### Complete genome of Thai PRRS viruses

During the 2001 PRRS outbreaks in Thailand, the PRRS viruses, 01CB1 and 01NP1 were isolated from the intensive swine farming areas. Additional 9 PRRS viruses isolated in Thailand from 2002-2008 were also included in the study (Table 1). To study the relationship and genetic characteristics of those Thai viruses, two isolates, "01CB1 and 01NP1", considering the Thai prototypes were selected for full-length genome sequencing since the pathogenicity of both viruses were previously studies. The viruses were identified as the EU (01CB1) and the US (01NP1) strains based on ORF 5 analysis [3]. In this study, we have elucidated the full-length sequences of PRRSV of 01CB1 containing 14,943 bp (52.67%GC) and 01NP1 containing 15,412 bp (52.76%GC). The viruses had untranslated regions (5' UTR and 3' UTR) and 8 open reading frames (ORFs) designated as ORF1a, ORF1b and ORF2-7. The details of genome organization of PRRS viruses, 01CB1 and 01NP1, were shown in table 2.

**Table 1:** List of PRRSV analyzed in this study

**Virus ID**	**Location**	**Year of isolation**	**Strain**	**GenBank accession number**
01CB1	Chonburi	2001	EU	DQ864705*
01NP1	Nakhon Pathom	2001	US	DQ056373*
02SB3	Saraburi	2002	EU	FJ908074
08RB103	Ratchaburi	2008	EU	FJ908075
08NP144	Nakhon Pathom	2008	EU	FJ908076
07NP4	Nakhon Pathom	2007	US	FJ908077
08NP147	Nakhon Pathom	2008	US	FJ908078
08NP148	Nakhon Pathom	2008	US	FJ908079
08RB51	Ratchaburi	2008	US	FJ908080
08RB154	Ratchaburi	2008	US	FJ908081
08RB160	Ratchaburi	2008	US	FJ908082

* nucleotide sequences of full length viruses

**Table 2:** Genome organization of PRRS viruses, 01CB1 and 01NP1, in this study

**ORFs**	**01CB1**			**01NP1**			Protein*
		
	**Nucleotides**		**Amino acid**	**Nucleotides**		**Amino acid**	
	**Position**	**Size**	**Size**	**Position**	**Size**	**Size**	
5' UTR	1-144	144	-	1-189	189	-	-
ORF1a	145-7335	7191	2396	190-7701	7512	2503	Replicase polyprotein; Nsp1alpha, beta (Papain-like cysteine protease); Nsp2 (cystein protease); Nsp3 - 8
ORF1b	7317-11708	4392	1463	7680-12071	4392	1463	RNA dependent RNA polymerase (Nsp 9 - 12)
ORF2	11719-12468	750	249	12073-12843	771	256	GP2 envelop protein
ORF3	12327-13124	798	265	12696-13460	765	254	GP3 envelop protein
ORF4	12869-13420	552	183	13241-13777	537	178	GP4 envelop protein
ORF5	13417-14022	606	201	13788-14390	603	200	GP5 envelop protein
ORF6	14010-14531	522	173	14375-14899	525	174	Matrix protein
ORF7	14521-14907	387	128	14889-15260	372	123	Nucleocapsid protein
3'UTR	14908-14943	36**	-	15261-15412	152	-	-

*Protein functions were identified based on Blast-P results and Wootton *et al*., 2000

** Poly A tail of 01CB1 was not identified in this study

### Phylogenetic analysis

**Figure 1:**
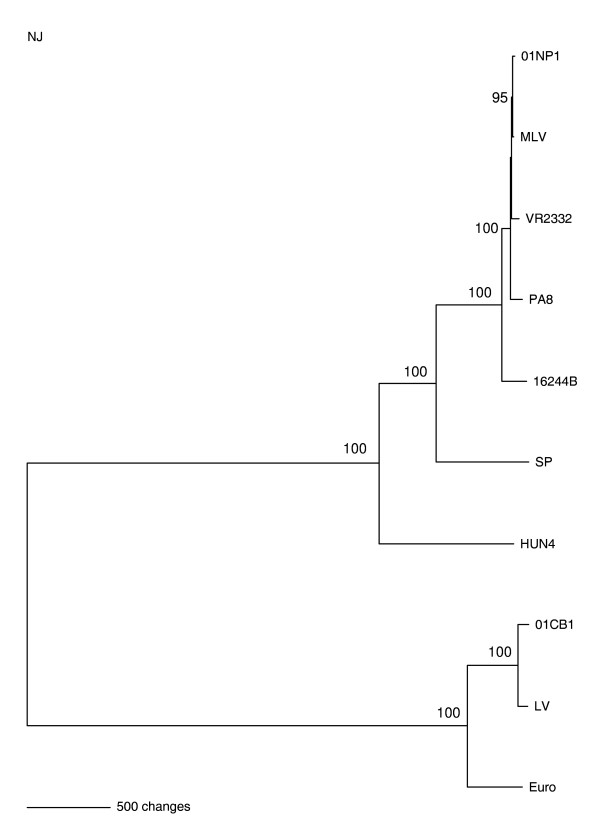
**Phylogenetic relationship of PRRS viruses, full length genome Sequences**. Whole genome sequences of ORF1-ORF7 were used for phylogenetic analysis using PAUP program applying NJ algorithm with distance setting of total character difference. Bootstrap analysis was conducted with 1000 replication.

**Figure 2:**
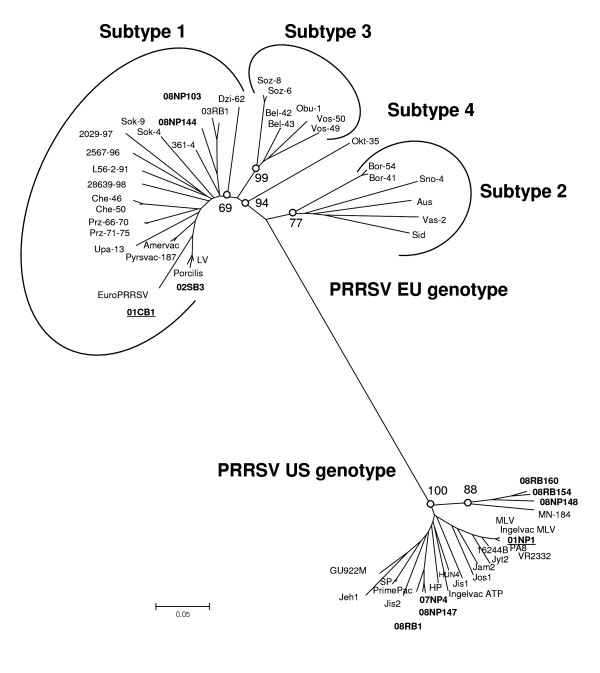
**Phylogenetic relationship of PRRS viruses, ORF 5 sequences**. ORF5 sequences were used for phylogenetic analysis using MEGA program applying NJ algorithm with Kimura 2-parameter. Bootstrap analysis was conducted with 2000 replication.

Phylogenetic analysis of the viruses showed that 01CB1 and 01NP1 were grouped into the separated lineages represented by the EU (LV and EuroRRSV) and US (MLV, VR2332, 16244B, PA8, SP and HUN4) isolates (Fig. 1). The 01CB1 was the most closely related to the LV virus (EU prototype), while the 01NP1 was the most closely related to the US MLV strain and the US prototype (VR2332). In general, phylogenetic analysis of full length sequences of PRRSV indicated that the Thai viruses were of both US and EU origin and exhibited the highest sequence similarity to those of EU prototype (LV) and the US prototype (MLV), respectively (Table 3). Phylogenetic analysis of ORF5 sequences of recent Thai PRRSV (2002-2008) were also analyzed, the results also showed high similarity among EU and US genotypes of the recent Thai PRRS viruses (2007-2008 viruses) with 01CB1 and 01NP1 (Fig 2). In addition, all EU isolates from Thailand (01CB1, 02SB3, 08RB103, 08NP144) are clustered into the EU genotype - subtype 1, which is the common subtype for EU-PRRSV worldwide as well as the EU vaccine strains.

**Table 3:** Pair-wise sequence comparison of full-length nucleotide sequences of PRRSV from Thailand and those of EU and US strains

**Pair-wise sequence comparison of PRRS viruses** (% nucleotide identity)*										
	01NP1	MLV	VR2332	PA8	16244B	SP	HUN4	01CB1	LV	EuroPRRSV
01NP1	100	99.9	99.5	99.3	98.5	93.2	89.1	59.1	59.1	59.2
MLV		100	99.7	99.4	98.6	93.2	89.2	59.1	59.1	59.2
VR2332			100	99.2	98.3	93.0	89.0	59.1	59.2	59.3
PA8				100	98.2	92.9	89.0	59.0	59.1	59.2
16244B					100	92.5	88.8	59.2	59.3	59.3
SP						100	88.3	59.1	59.1	59.1
HUN4							100	59.4	59.5	59.5
01CB1								100	99.2	95.2
LV									100	95.3
EuroPRRSV										100

* Sequence comparison of PRRSV in this study was based on ORF1-ORF7 sequences.

### Genetic analyses

Pair-wise sequence comparisons of full-length sequences of the Thai isolates are presented in table 3. The two Thai isolates were compared with eight representative PRRS viruses of both genotypes obtained from the GenBank database that had been completely sequenced. The 01CB1 displayed the highest percentage of nucleotide identity to the EU isolates (LV and EuroPRRSV), while the 01NP1 had high percentage of similarity to the US isolates (MLV, VR2332, 16244B, PA8, SP, HUN4) with more than 98% nucleotide identity. However, the percent homology between the two Thai isolates, 01CB1 and 01NP1, was 59.81%.

In this study, 5' UTR of 01NP1 and 01CB1 had 189 bp and 144 bp in length. 5'UTR of 01CB1 was almost identical to the 5'UTR of VR2332 (99.5%) and the US-MLV (98.6%) (data not shown), while 5' UTR of 01NP1 displayed profound nucleotide sequence identity (more than 90%) with the US isolates. Similar findings were also observed in 3' UTR that 01CB1 and 01NP1 shared high percentage of nucleotide identity of 3' UTR of the EU and US genotypes.

**Figure 3:**
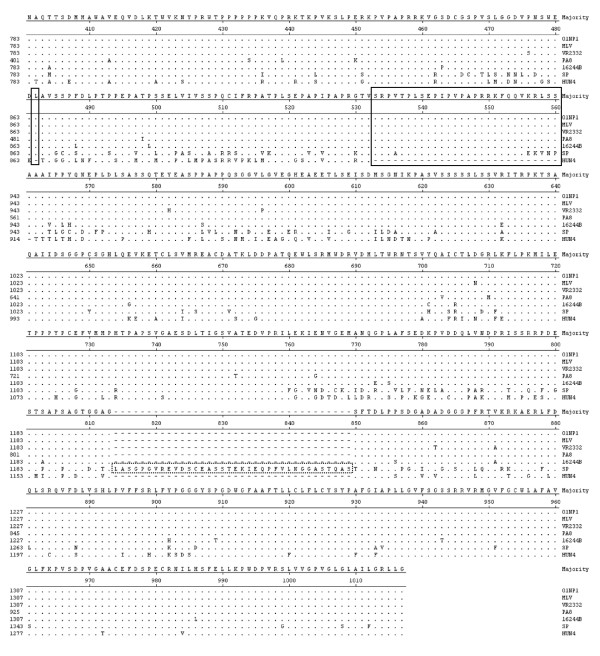
**Sequence alignment of NSP2 of PRRSV viruses (US strains)**. Deduced amino acids of NSP2 gene of 01NP1 were compared to those of US strains (MLV, VR2332, PA8, 16244B, SP and HUN4). No amino acid deletions position 482 and 533-561 were found in most US strain except HUN4 (solid blocks). While 36 amino acid insertions were observed in SP (Asian vaccine strain) (dotted block).

ORF1a and ORF1b of 01CB1 and 01NP1 encoded proteins of 2,396 and 1,463 and 2,503 and 1,463 amino acids, respectively. ORF1a and ORF1b of 01CB1 were similar to those of the EU strains (99.2% and 99.3%) (data not shown). Comparison of deduced amino acids revealed that ORF1a had more polymorphic sites than ORF1b proteins. Polymorphic sites in ORF1a of 01CB1 and 01NP1 were 125/2396 and 61/2503. On the other hand, polymorphic amino acids in ORF1b were 27/1463 (01CB1) and 16/1463 (01NP1). These findings indicated that ORF1a was continuously changing and evolving as previously described especially in the Nsp2 region [8]. In this study, deduced amino acids of the Nsp2 proteins of 01NP1 were compared to those of US strains (MLV, VR2332, PA8, 16244B, SP and HUN4) (Fig 3). Amino acid deletions were found at position 482 (1 aa) and 533-561 (28 aa) in Nsp2 of the Chinese isolate (HUN4) but not in other US strains. Thirty six amino acid insertions were also observed in the Asian vaccine strain (SP), but not found in 01NP1 Thai isolate.

**Figure 4:**
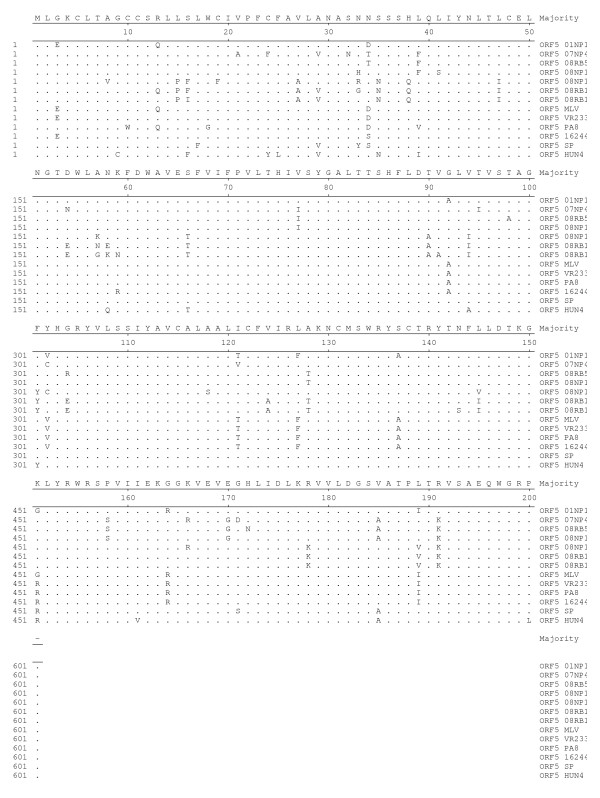
**Sequence alignment of ORF5 of PRRSV viruses (US)**. Deduced amino acids of ORF5 gene of 01NP1 and 6 PRRSV were compared to those of US strains (MLV, VR2332, PA8, 16244B, SP and HUN4).

**Figure 5:**
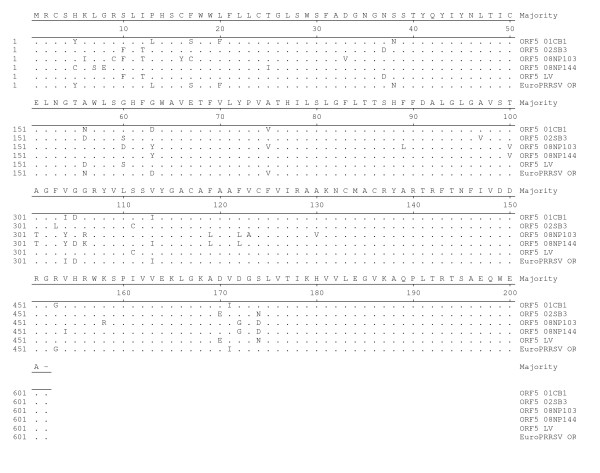
**Sequence alignment of ORF5 of PRRSV viruses (EU)**. Deduced amino acids of ORF5 gene of 01CB1 and 3 PRRSV were compared to those of EU strains (LV and EuroPRRSV).

ORFs2-7 genes encoded structural proteins of the PRRSV including envelop protein (ORF2-5), matrix protein (ORF 6) and nucleocapsid protein (ORF 7). Structural genes of 01CB1 and 01NP1 were approximately 3 kb in size. In this study, ORF 2-7 of the two viruses were conserved (less polymorphic sites). ORF 2-7 of 01CB1 and 01NP1 were similar to ORF2-7 of the LV and the US-MLV viruses (>99.0% identity) (Data not shown). Out of 5 structural genes (ORFs2-7), ORF 7 was highly conserved in both EU and US strains, while ORF5 was less conserved among both strains. Deduced amino acids of ORF5 gene of 01NP1 and 01CB1 and additional 9 PRRSV (2002-2008) were compared to those of US strains (MLV, VR2332, PA8, 16244B, SP and HUN4) and EU strain (LV and EuroPRRSV) (Fig 4 and 5). The results showed that the US strains (01NP1) had 11 polymorphic sites comparing to US consensus and the EU strains (01CB1) had 13 polymorphic sites comparing to the EU consensus (Fig 4 and 5). Interestingly, polymorphic sites in ORF5 of EU strains were found more than those of US strains. Sequence distances of ORF5 among PRRS viruses are 82.0-99.5% (among US genotype) and 84.7-99.5% (among EU genotype). These findings indicated moderate genetic diversity among Thai PRRSV in both genotypes.

In summary, genetic analyses of untranslated region (5' UTR and 3' UTR) and ORF 1-7 showed that 01CB1 was mostly similar to the EU prototype, LV (98.5% -99.7%). The Thai US strain, 01NP1 was closely related to the US-MLV vaccine strain (99.4%-100%).

## Discussion

In this study, we reported full-length sequences of the Thai PRRS viruses of both EU (01CB1) and US (01NP1) genotypes. The full-length size of the EU strain, 01CB1, is 14,943 bp, similar to the two EU strains (LV; 15,101 bp and EuroPRRSV; 15,047 bp) [5,11]. On the other hand, 01NP1 isolate has 15,412 bp in size. The size of this virus is similar to the US strains (VR2332, 15,411 bp and 16244B, 15,411 bp) [4,6], the US-MLV vaccine (MLV, 15,412 bp) [7], Canadian field strain (PA8, 15,411 bp) [8], Asian vaccine strain (SP, 15,520 bp) [9] and Asian field strain (BJ-4, 15,410 bp and HUN4, 15352 bp) [10].

Genome organization of the Thai PRRS viruses contained 8 open reading frames. Two non structural genes, ORF1a and ORF1b, composed 70% in size of the genome. ORF1a and ORF1b encoded replicase polyproteins, which subsequently cleaved to 13 subunits (Nsp1a/b-Nsp12). ORF2-7 were structural genes that encode envelop protein (ORF2-5), matrix protein (ORF 6) and nucleocapsid protein (ORF7) [8]. Most full-length sequences of PRRSV had identified of 8 ORFs in the genome, however some studies have reported additional ORF2 (ORF2a and ORF2b) encoding unknown protein function.

Comparison of full-length sequences of the Thai PRRS viruses (01CB1 and 01NP1) with other PRRS viruses from the European, north American and Asian countries revealed that 01CB1 virus was similar to the EU strains especially the EU prototype, Lelystad (99.2%). Unexpectedly, 01NP1 had nucleotide sequences similar to the US-MLV (99.9%) and the US-prototype (VR2332) (99.5%). Phylogenetic analysis showed that 01CB1 and 01NP1 were clustered into the EU and the US lineages, respectively. 01CB1 was closely related to the LV virus, the EU prototype whereas, 01NP1 was closely related to the US-MLV (vaccine strain) and VR2332 (US prototype). Our results indicated that the Thai EU strain evolved from the LV. The introduction of the Thai EU strain of PRRSV may possibly due to the importation of persistently infected pigs or semen. Interestingly, 01NP1 was closely related to the US-MLV vaccine strain. Since the US-MLV vaccine was not available in Thailand until 2005, the contaminated vaccine-like virus might have persisted in the imported pigs or semen at that time. Our previous report found that the Thai EU isolates were closely related to the Danish viruses and the Thai US isolates were closely related to the Canadian viruses [3]. Since the US-MLV has been found in the Danish pig population [18] at the same time of the first PRRSV report in Thailand [2]. 01NP1 might originate from persistently US-MLV infected imported pigs either from Canada or Denmark. Similarly, the evidence of the field strain (PA8) that originated and evolved from the US-MLV vaccine strain (RespPRRS) had been documented in Canada [8]. Unfortunately, full length nucleotide sequence of the Spanish vaccine virus (Amervac) was not available for analysis since only the Spanish vaccine was the only live vaccine available at that time. The analysis will rule out the possibility that the Thai EU strain may also evolve from imported Spanish vaccine strains in the 90s.

In this study, the most variable ORFs were ORF1a (Nsp2) and ORF5. Both ORF1a and ORF5 were previously reported as highly variable regions. ORF1a (Nsp2 subunit) can be used as genetic marker for monitoring the mutation or genetic changes as well as for differential diagnosis of PRRS viruses [9,19,20]. Recently, atypical PRRS outbreaks have been reported in China since 2006 causing severe economic losses in the Chinese swine industry. Genome analysis of the Chinese viruses revealed that the viruses contain 2 distinct amino acid deletions in the Nsp2 gene indicating highly virulent of PRRS viruses [10,12]. The multiple deletions in this specific Nsp2 region reported in the Chinese isolates causes the so-called 'Swine high fever syndrome' [10]. The pathogenesis of turning virulence of the Chinese viruses is still unclear and needed to be elucidated. Fortunately, we did not see any deletion in our Thai isolates similar to the Chinese viruses.

Similar to other studies, the variation of ORF5 region can be applied for identification and differentiation of the PRRSV. In addition, ORF5 can also be used for the study of genetic diversity of the viruses [3,17,21,22]. In this study, 9 additional PRRS viruses were analyzed in the ORF5 region. Phylogenetic analysis of ORF5 clearly separated US genotype and EU genotype, which both genotypes can be found circulating in Thailand (Fig 2). It has been known that the US genotype is more diverse than the EU genotype. However, in this study, all Thai EU-genotype isolates are more diverse and belonged to the EU genotype-Subtype 1, similar to some European isolates (The Netherlands, Denmark, Spain, Poland and Italy) but not the PRRSV from Eastern European which are belonged to EU genotype-Subtype 2, 3 and 4 (Belarus and Lithuania) [23]. Currently, both EU and US genotypes are still circulating in the Thai swine industry with predominantly the US genotype (data not shown). Interestingly, our results indicated that all Thai PRRS viruses in this study had evolved from the Thai PRRSV prototypes of both genotypes. No evidence of recent imported new PRRSV strains was found in this study possibly due to the Department of Livestock Development, Thailand do not allow the importation of PRRSV-positive animals.

## Conclusion

In conclusion, our study provided full-length genome sequences of the Thai PRRS viruses of both genotypes. The genetic and cluster analysis of the Thai PRRSV of the EU genotype (01CB1) may evolve from the EU prototype, the Lelystad virus. On the other hand, the Thai PRRSV of the US genotype (01NP1) may originate and evolve from the US-MLV vaccine virus or its derivatives. It should be noted that ORF1a (Nsp2) and ORF5 contained highly variable regions and can be used as diagnostic markers for prevention and control of newly emerged PRRSV. This work highlights the significance of full-length sequences of PRRSV in Thailand for future studying of the genesis and evolution of the PRRS viruses.

## Methods

### PRRS viruses

The Thai EU isolate (01CB1) used in this study was isolated from the nursery pigs having PRDC problem in Chonburi province, the Eastern region of Thailand in 2001. The EU PRRSV caused reproductive failure in a 3,000 sow herd and later the respiratory disease with moderate morbidity and mortality in the nursery pigs. The Thai US isolate (01NP1) was isolated in Nakhon Pathom province located in the central region of Thailand in the same year from the nursery pigs in a 2,000 sow herd with more than 10% loss after weaning. Both farms are practicing a continuous-flow system and piglets are weaned weekly. The pathogenesis study of the 01CB1 and the 01NP1 virus was done and the 01CB1 was identified as a low virulence strain while the 01NP1 was identified as a high virulence strain. Based on ORF5 sequence analysis, 01CB1 and 01NP1 were characterized and grouped in the EU and the US genotypes respectively [3]. Additional 9 PRRS viruses isolated in Thailand from 2002-2008 were included in the study (Table 1). The viruses were later identified as EU genotype (n = 3) and US genotype (n = 6) based on ORF5 nucleotide sequencing and then include in the phylogenetic analysis.

### Virus isolation

Virus isolation was done from the lung tissues as previously described [24]. The cell culture-adapted viruses were propagated in MARC-145 cells in minimum essential medium (MEM) (Hyclone, USA) with 5% fetal calf serum (FCS) (Hyclone, USA) for 3 passages. Immunoperoxidase monolayer assay (IPMA) using SDOW-17 was used to confirm the presence of PRRS virus [3]. The virus concentration of 10^3 ^TCID50/ml was used for viral RNA preparation in this study.

### Viral RNA and cDNA preparation

RNA isolation using QIAamp RNA Mini Kit (Qiagen, Hilden, Germany) was done on the stock virus solution following the manufacture's instruction. In brief, 200 μl of virus-containing supernatant was mixed with 200 μl buffer AVL and incubated for 10 minutes. Then, 500 μl of ethanol was added to the mixture. The mixture was then transferred to QIAamp spin column and centrifuged at 8000 rpm for 2 min. The spin column was subsequently washed with 500 μl of buffer AW1 and AW2 and centrifuged at 8000-14000 rpm for 3 min. Finally, 50 μl of buffer AVE was added and centrifuged at 8000 rpm for 3 min to elute viral RNA. cDNA synthesis was then performed by incubating viral RNA with 0.5 μg Random primers (Promega, Madison, WI) at 70°C for 5 min and then 4°C for 5 min. The mixture was then added with 1× Improm-II reaction buffer (Promega), 0.5 mM dNTPs (Fermentus), 2.5 mM MgCl2 (Promega), 10 U of Rnasin Ribonuclease inhibitor (Promega) and 1 μl of Improm-II Reverse Transcriptase. The mixture was incubated in thermal cycler at 25°C for 5 min, 42°C for 60 min and 70°C for 15 min.

### PRRSV genome sequencing

Oligonucleotide primers used in this study were designed based on sequence information of the EU and US prototypes, LV and VR2332, respectively. Additional primers were designed for gap closure to complete whole genome sequences of the viruses. Sequence information of each oilgonucleotide primers are provided in additional file 1. PCR amplification of viral RNA was performed as previously described [3]. In brief, 25 μl of PCR reaction was prepared by adding 2 μl of cDNA, 1× Eppendrof Master Mix (Eppendrof, Hamburg, Germany) and 0.8 μM of oligonucleotide primer. The PCR reaction mixture was incubated in thermal cycler with condition: 95°C for 10 min and 35 cycle of denaturation (95°C for 45 Sec), annealing (55°C for 45 Sec), extension (72°C for 90 Sec), and final extension of 72°C for 15 min. The PCR products were then analyzed in 2% gel electrophoresis (FMC Bioproducts, Rockland, ME). The PCR products were then purified using the Perfectprep Gel Cleanup Kit (Eppendorf,) for further DNA sequencing. The DNA sequencing reaction was performed, using a commercially available kit (Big Dye Terminator V.3.0 Cycle Sequencing Ready Reaction; Foster City, CA), at a final volume of 20 μl, containing 8 μl of dye terminator and 12 μl of specific sequencing primer at the concentration of 3.2 pmol. The sequencing products were analyzed with the ABI-Prism 310 Genetic Analyzer (Perkin Elmer, Norwalk, CT). ORF5 nucleotide sequencing was also performed using the oligonucleotides specific for ORF5 and then subjected for DNA sequencing. At least 4 coverages of viral nucleotide were performed in the study, to ensure the quality of PRRS genome sequencing.

### Analysis of nucleotide and amino acid changes in PRRS viruses

Genome assembly was conducted by using a computer program SeqMan (DNASTAR, Madison, WI). In this study, the full-length genome sequencing of 2 viruses (01CB1 and 01NP1) was conducted to reach at least 4 time coverages of each virus. In addition, the chromatograms of nucleotide sequences of each PCR products were rechecked and validated to ensure the type and position of nucleotide and amino acid changes in PRRS genome. The sequence alignment and amino acid comparison were done by computer program, MegAlign (DNASTAR). The phylogenetic analysis was performed using the PAUP version 4.0 software (Sinauer Associates, Sunderland, MA) applying NJ algorithm with distance setting of total character difference and the MEGA3 software applying NJ algorithm with Kimura 2- parameter. Bootstrap analysis was conducted with 1000 replications. The nucleotide sequences of the Thai PRRS viruses, 01CB1 and 01NP1 were submitted to the Genbank database under the accession numbers: [01CB1: DQ864705 and 01NP1: DQ056373]. The ORF5 nucleotide sequences of 9 viruses were also in the Genbank database under the accession numbers: [FJ908074-FJ908082].

## Competing interests

The authors declare that they have no competing interests.

## Authors' contributions

AA carried out experimental design, genome sequencing, genetic and cluster analysis and drafting the manuscript and final approval. RK conducted virology and molecular cluster analysis studies. SP conducted whole genome and gene sequencing studies. PW conducted molecular genetic work. SS and RT participated in virology study and drafting the manuscript. All authors read and approved the final manuscript.

## Supplementary Material

Additional file 1 **Oligonucleotide primers used in the study**. List of oligonucleotide primers used in this study. Click here for file
