# Supplementary material for: Comparative analysis of complete nucleotide sequence of porcine reproductive and respiratory syndrome virus (PRRSV) isolates in Thailand (US and EU genotypes)
Source: Virol J. 2009 Sep 16;6:143. doi: 10.1186/1743-422X-6-143 (PMC2753317; doi:10.1186/1743-422X-6-143)
Supplement: Additional file 1 — Oligonucleotide primers used in the study. List of oligonucleotide primers used in this study. [file 1743-422X-6-143-S1.PDF]

Additional file 1: List of oligonucleotide primers used in this study.

Fragment	Name	**Sequence**	bp	Expected size
**EU strain**				
Primer Set1 [a]				
52-1255	EU1-F	5’-ACCTTGGAGGCGTGGGTAC	19	
	EU1-R	5’-TGCCTGATCCAAGACTGGG	19	1204
1030-2251	EU2-F	5’-ACGTGTTTGATGGCAAGTGC	20	
	EU2-R	5’-GGTGAACACAGGACAACAGCA	21	1222
2061-3276	EU3-F	5’-GATGTTGAAGGATTGTCCGAGC	22	
	EU3-R	5’-GAGGCGGTGTGTCTTGAATCA	21	1216
3060-4264	EU4-F	5’-GCCGATGTCCATGCAAAAAT	20	
	EU4-R	5’-GCTATGGGCTTTTGGTGTGG	20	1205
4046-5294	EU5-F	5’-GCGTTGTCACAAGTGTTGGG	20	
	EU5-R	5’-AAGGGATCCAACTGCAGACG	20	1249
5072-6321	EU6-F	5’-CGGCATCCTCTCACTAGGGA	20	
	EU6-R	5’-CGAGGGTATGGAGTCCACCA	20	1250
6118-7349	EU7-F	5’-TCACGGCATCTCTCAACCG	19	
	EU7-R	5’-TTCCACCTCGGCAGCTGTA	19	1232
7126-8392	EU8-F	5’-ATGAGGGTGTGCAAACCACC	20	
	EU8-R	5’-CCAATTCTCCTTGACAGCGC	20	1267
8102-9350	EU9-F	5’-CATGGCACTTCCAAGGCTG	19	
	EU9-R	5’-CGCAGTGGCGGAATTTCTT	19	1249
9190-10406	EU10-F	5’-TGTGCTTGCATTGACCATGA	20	
	EU10-R	5’-TGGCTACAAGTGCTCGGGA	19	1217
10188-11417	EU11-F	5’-TCCAGCCTTGTTACAGGGAGA	21	
	EU11-R	5’-CCGGTCCTATACACGGATCAA	21	1230
11190-12399	EU12-F	5’-TCACCGATGTGTACCTCCCC	20	
	EU12-R	5’-TGAAATCGGTCAACTTGGGC	20	1210
12243-13483	EU13-F	5’-GCAGTGGAGGCGGATTCTT	19	
	EU13-R	5’-GAATGGCGAACAAACAAGCA	20	1241
13234-14479	EU14-F	5’-AACGCGGACCTGCTGATG	18	
	EU14-R	5’-TGCTCGGTTACCAGACGCT	19	1246
14281-15062	EU15-F	5’-ATCCACCAACCGTGTCGC	18	
	EU15-R	5’-ACCCCCATGTGATCGCC	17	782
Primer Set 2 [b]				
2230-2970	EU 2230	5’-ACCGGATGCAAAAGAGTTCG	20	
	EU 2970	5’-GTCAACCACCGGAGCATCTT	20	691
3240-4070	EU 3240	5’-CTGGTGGCACAGTGGGATAG	20	
	EU 4070	5’-GAAAGGGCCAAATCTGCAAG	20	698

4230-5120	EU 4230	5’-CCATCCATCAACCACACCAA	20	
	EU 5120	5’-TGCCCAAAGAAGGCCAGTTA	20	878
5330-6130	EU 5330	5’-GGAGGGGTTTTCACCATTGA	20	
	EU 6130	5’-CCCATGCAAGCACAAAGAGT	20	697
6550-7130	EU 6550	5’-CGGCCAGTACATTGAAGCAG	20	
	EU 7130	5’-GTACCGGGAATCATCCGTGT	20	496
7400-8200	EU 7400	5’-TTGTGACTGAAACGGCGGTA	20	
	EU 8200	5’-TCCTTGGGTGGATAGGTCGT	20	710
9400-10200	EU 9400	5’-CCTCTTGATGCCGTGCTAAA	20	
	EU 10200	5’-TAGGAATCAAAGCCGACAGG	20	596
10520-11050	EU 10520	5’-TGCGGATAATGCAGTCACAAC	21	
	EU 11050	5’-GTGGGGGAGTTCTTTTGCTG	20	485
11370-12150	EU 11370	5’-CCCAAGGATGCCGTTGTAT	19	
	EU 12150	5’-TGGTCTGGTAAATGCGACGA	20	767
12800-13180	EU 12800	5’-GGACAAGCGACACCAGTTCA	20	
	EU 13180	5’-TGGGCCACATAATCTGTGAAA	21	557
13960-14350	EU 13960	5’-TGGTAGAAAAATTGGGCAAAGC	22	
	EU 14350	5’-TGAAGCTGTAAACACCCCACA	21	371
Primer Set 3 [C]				
1-239	PRRS EU 1	5'-ATGATGTGTAGGGTATTC	18	
	PRRS EU 239	5'-GGAGAACGTCCCAGACAT	18	239
14990-15115	PRRS EU 14990	5'-TCTACATCCGCCAGTCAG	18	
	PRRS EU 15115	5'-AATTTCGGTCACATGGTTC	19	126
**US strain**				
Primer Set 4 [d]				
0-1203	PRRS-1F	5’ATGCATGCTAATACGACTCACTATAG CGCCCGGGCAGGTGTTG -3’	47	1202
	PRRS-1R	5’-GCGGATCCAACTCCCTTAACGG -3’	22	
1150-2191	PRRS-2F	5’-CTAAACGGACCTATCGTCG -3’	19	1040
	PRRS-2R	5’-AGGTGTCGATTACGCGTGGC -3’	20	
2103-3212	PRRS-3F	5’-GTTTGACCTGTACCTCCGTGG -3’	21	1108
	PRRS-3R	5’-CTGCTTGATGACACGGACG -3’	19	
3135-4741	PRRS-4F	5’-GCATGAAGCTGAGGAAACC -3’	19	1605
	PRRS-4R	5’-ATGGAACAGCGGAAACCTTGACC -3’	23	
4329-6293	PRRS-5F	5’-CTGTATCTTGGCTGGAGCTTACGTGC -3’	26	1963
	PRRS-5R	5’-GCATGTCCCATCATTCTCCACAGG -3’	24	
6193-7630	PRRS-6F	5’-CTTTGTGCCTTGCTTGCTGCC -3’	21	1437
	PRRS-6R	5’-CTTTGGCAGTCAGTTCGC -3’	18	
7550-10977	PRRS-7F	5’-GAGTTCAATGGGAAGCTGC -3’	19	3426
	PRRS-7R	5’-AGTTGTGTGCGACCTTGG -3’	18	
10630-12209	PRRS-8F	5’-CATTCGATGTGGTTACATTGCATTTGCCC -3’	29	1578
	PRRS-8R	5’-CCAACCGGCGATGGTGAAGC -3’	20	

12073-14914	PRRS-9F	5'-CGGATCCATGAAATGGGGTCCATGCA -3'	26	2841
	PRRS-9R	5'-GTCTGCTTGCCGTTGTTA -3'	18	
14365-end	PRRS-10F	5'-CGGATCCCAGCGGAACAATGGGGT-3'	24	1046
	PRRS-10R	5'-TTCTAGAATTCAGCGGCCGC$(T)_{30}N_{-1}$N-3'	50	
Primer Set 5 [e]				
1-1324	PRRS-12-F	5'-GGTGTTGGCTCTATGCCTTG -3'	20	
	PRRS-1271-R	5'-GGCTCAACCCTTATTCTGAGG -3'	21	1260
2000-3400	PRRS-2001-F	5'-CAAGTGCGCTTAGGGAAAAT -3'	20	
	PRRS-3390-R	5'-GAGAAAGCCATTCCTGCGTA -3'	20	1390
3200-4700	PRRS-3216-F	5'-TCCTTGTCCAGCGTGAGAAT -3'	20	
	PRRS-4577-R	5'-GGGTTTTTCAGAGGGTTGCT -3'	20	1362
4440-5800	PRRS-4456-F	5'-CGTCACTTATCGACCTGTGC -3'	20	
	PRRS-5743-R	5'-GTAAGGACATGTGCGGCAGT -3'	20	1288
5450-6900	PRRS-5525-F	5'-GCACCAGATGGGACCTACTT -3'	20	
	PRRS-6842-R	5'-GGAAATCCAAGTCCTCGTCA -3'	20	1318
6700-7900	PRRS-6700-F	5'-GAGGGAAAGTTGAGGGAAGG-3'	20	
	PRRS-7900-R	5'-GTGCTCAACCGCGTCTTT-3'	18	1146
7750-9000	PRRS-7750-F	5'-TGGCCAGTGAGGTTGAGCTA -3'	20	
	PRRS-9000-R	5'-TTCAGCACGTACGACGGTAG -3'	20	1251
8900-10100	PRRS-8900-F	5'-GCAATTGTCCGCTGGTTT -3'	18	
	PRRS-10100-R	5'-GCCAGTATGTTTTCCCAGCA -3'	20	1192
11100-12100	PRRS-11100-F	5'-GGTGCCGGCTATATGGTG -3'	18	
	PRRS-12100-R	5'-TCAGGCCTAAAGTTGGTTCAA -3'	21	931
11250-13150	PRRS-11250-F	5'-GCCTGAATTGAAATGAAATGG -3'	21	
	PRRS-13150-R	5'-GAGGCAAGGTGGTGTTCTG -3'	19	1104
13100-14200	PRRS-13100-F	5'-GGGCAGAACACCACCTTG -3'	18	
	PRRS-14200-R	5'-ACGGAACCATCAAGCACAAC -3'	20	1199
14100-15400	PRRS-14100-F	5'-GGCTGCGTTGACTTGCTT -3'	18	
	PRRS-15400-R	5'-GGCACAATGTCAATCAGTGC -3'	20	1198
Primer Set 6 [f]				
5' UTR	PRRS-1F	5'-ATGACGTATAGGTGTTGGCTCT-3'	22	400
	PRRS-400R	5'-AGCACTCAACAGTGGGGAAT-3'	20	
3' UTR	ORF7-F	5'-CGGATCCCCTTGTCAAATATGCCAA-3'	25	565
	3' UTR	5'-TTAATTTCGGCCGCATGGTTCT-3'	22	

[a]: specific primers (set 1) were designed based on sequence information of Lelystad virus

[b]: specific primers (set 2) were designed for gap closure based on available sequences and Lelystad virus

[C]: specific primers (set 3) were designed to amplify 5' UTR and 3' UTR

[d]: specific primers (set 4) were provided by Wootton *et al.*, 2000

[e]: specific primers (set 5) were designed for gap closure based on available sequences and VR2332

[f]: specific primers (set 6) were designed to amplify 5' UTR and 3' UTR
